# Impact of Frontline Treatment Strategies on Outcomes in Patients With Acute Myeloid Leukemia, Myelodysplasia‐Related

**DOI:** 10.1002/cnr2.70607

**Published:** 2026-06-21

**Authors:** Yi Li, Dian Jin, Peiying Fang, Xinyi meng, Donghua He, Jintao Lin, Jing Le, Wenxiu Shu, Qianqian Yang, Jingsong He, Zhen Cai

**Affiliations:** ^1^ Bone Marrow Transplantation Center The First Affiliated Hospital, Zhejiang University School of Medicine Hangzhou China; ^2^ Liangzhu Laboratory, Zhejiang University Medical Center Hangzhou China; ^3^ Institute of Hematology, Zhejiang University Hangzhou China; ^4^ Zhejiang Province Engineering Laboratory for Stem Cell and Immunity Therapy Hangzhou China; ^5^ The Affiliated Li Huili Hospital of Ningbo University Ningbo China

**Keywords:** acute myeloid leukemia, AML‐MR, chemotherapy, myelodysplasia‐related changes, prognosis, venetoclax

## Abstract

**Background:**

The 5th edition of the World Health Organization (WHO) classification updated the term for acute myeloid leukemia with myelodysplasia‐related changes (AML‐MRC) to acute myeloid leukemia, myelodysplasia‐related (AML‐MR), along with new diagnostic criteria. However, the clinical prognostic outcomes of patients diagnosed with AML‐MR based on the new WHO definition remain poorly defined, and the optimal frontline treatment strategy for this patient population has not been fully clarified.

**Aims:**

This study aimed to compare the clinical efficacy and survival outcomes of three mainstream frontline treatment regimens for newly diagnosed AML‐MR patients and identify independent prognostic factors affecting patient survival.

**Methods and Results:**

We collected data from 142 patients with newly diagnosed AML‐MR and divided them into three cohorts depending on different frontline therapies: low‐intensity chemotherapy (LIC), intensive chemotherapy (IC), and venetoclax plus hypomethylating agent (VEN/HMA). The overall response rate in the VEN/HMA cohort was higher than in the LIC or IC cohort (VEN/HMA vs. LIC, 72.7% vs. 42.1%, *p* = 0.010; VEN/HMA vs. IC, 72.7% vs. 54.3%, *p* = 0.049). Event‐free survival (EFS) in the LIC cohort was inferior to that in the IC cohort (median 2.5 vs. 4.8 months, *p* = 0.037) or VEN/HMA cohort (median 2.5 vs. 6.4 months, *p* = 0.006). The overall survival (OS) in the LIC cohort was inferior to that in the IC cohort (median 11.9 vs. 19.4 months, *p* = 0.009). No statistically significant differences in EFS or OS were observed between the IC and VEN/HMA cohorts. Multivariate analyses showed that prior HMA exposure and frontline LIC therapy were associated with inferior EFS, and allogeneic hematopoietic stem cell transplantation was associated with better EFS and OS.

**Conclusion:**

Our study suggests that in patients with AML‐MR, frontline therapy with VEN/HMA or IC improves outcomes compared with LIC. Allogeneic hematopoietic stem cell transplantation is encouraged for all eligible patients with AML‐MR.

**Abbreviations**
allo‐HSCT: allogeneic hematopoietic stem cell transplantation
AML: acute myeloid leukemia
AML‐MR: acute myeloid leukemia, myelodysplasia‐related
AML‐MRC: acute myeloid leukemia with myelodysplasia‐related changes
CI: confidence interval
CR: complete remission
CRi: CR with incomplete recovery of blood counts
EFS: event‐free survival
ELN: European LeukemiaNet
HMA: hypomethylating agent
HR: hazard ratio
IC: intensive chemotherapy
LIC: low‐intensity chemotherapy
MDS: myelodysplastic syndrome
MDS/MPN: myelodysplastic/myeloproliferative neoplasms
MLFS: morphologic leukemia‐free state
MRD: measurable residual disease
ORR: overall response rate
OS: overall survival
VEN: venetoclax
WHO: World Health Organization

## 1 Introduction

Acute myeloid leukemia (AML) is a heterogeneous hematological malignancy with different biological and clinical characteristics [1]. Based on the morphologic, immunophenotypic, molecular, and cytogenetic data, AML is classified into different subtypes [2]. AML with myelodysplasia‐related changes (AML‐MRC) is a subtype that accounts for 25%–34% of all AML cases and is more common in elderly patients, with a median age of 73 years [3, 4]. In previous studies, the prognosis of AML MRC was shown to be worse than that of AML non‐MRC, with reduced complete remission (CR) rates and overall survival (OS) [5]. In addition, it usually responds poorly to standard intensive induction chemotherapy [5].

In 2022, the 5th edition of the World Health Organization (WHO) classification updated the definition of AML‐MRC to AML, myelodysplasia‐related (AML‐MR). AML‐MR is defined as a neoplasm with ≥ 20% blasts expressing a myeloid immunophenotype and harboring specific cytogenetic and molecular abnormalities associated with myelodysplastic syndrome (MDS), arising de novo or following a known history of MDS or myelodysplastic/myeloproliferative neoplasms (MDS/MPN) [2]. Key changes include: removal of morphology alone as a diagnostic premise to make a diagnosis of AML‐MR; update of the defining cytogenetic criteria; and introduction of a mutation‐based definition based on a set of eight genes—SRSF2, SF3B1, U2AF1, ZRSR2, ASXL1, EZH2, BCOR, STAG2, > 95% of which are present specifically in AML arising post‐MDS or MDS/MPN [2, 6, 7]. The clinical outcomes for patients with the newly defined AML‐MR are not very clear. Given that the US Food and Drug Administration has recently approved CPX‐351, a liposomal formulation of daunorubicin and cytarabine [8], or venetoclax, a BCL2 inhibitor [9] for AML, it is necessary to determine the optimal frontline treatment strategies for patients with AML‐MR. Here, we retrospectively evaluated 142 patients with newly diagnosed AML‐MR and evaluated the impact of clinical features and treatment strategies on the outcomes.

## 2 Methods

### 2.1 Study Design and Participants

This was a retrospective, two‐center cohort study conducted at the First Affiliated Hospital of Zhejiang University School of Medicine and the Affiliated Li Huili Hospital of Ningbo University. Adult patients (age ≥ 18) with newly diagnosed AML‐MR at the two centers between January 2019 and June 2023 were reviewed. The diagnostic criteria for AML‐MR were according to the 5th edition of the WHO classification, mentioned above [2]. Patients were divided into three cohorts for analysis, depending on the frontline therapies they received: [1] low‐intensity chemotherapy (LIC) without venetoclax, [2] intensive chemotherapy (IC) without venetoclax, and [3] venetoclax plus hypomethylating agent (VEN/HMA) without a third agent. HMAs included azacytidine and decitabine. LIC included low dose cytarabine ± low dose daunorubicin/idarubicin/homoharringtonine/aclacinomycin, with or without HMAs. IC included cytarabine + daunorubicin/idarubicin and cytarabine + homoharringtonine ± aclacinomycin, with or without HMAs. Patients were required to accept at least one cycle of therapy and follow up to a response assessment or death. Patients who had received venetoclax‐based therapy, IC, or allogeneic hematopoietic stem cell transplantation (allo‐HSCT) for MDS or MDS/MPN prior to the diagnosis of AML‐MR were excluded. Because very few patients received single‐agent HMAs as induction therapy, we also excluded this population of patients from analysis. Finally, 126 patients from The First Affiliated Hospital of Zhejiang University School of Medicine and 16 patients from the Affiliated Li Huili Hospital of Ningbo University were included in this study.

### 2.2 Baseline Data Collection

Patient DNA was extracted from the bone marrow collected at diagnosis according to the instructions of the Genomic DNA Whole Blood Extraction kit (Concert Bioscience, China). Gene library amplification was performed by a KAPA Hyper Prep Kit (Roche, Switzerland). The targeted 78 gene sequencing panel was provided by Acornmed Biotechnology Co. Ltd. Multiplex libraries were sequenced via the Illumina NovaSeq instrument. This panel fully covers the eight genes required for the WHO 5th edition diagnosis of AML‐MR and the genes required for European LeukemiaNet (ELN) 2022 risk categories [10], with a detection sensitivity of 0.01%. The intrabatch and interbatch repeatability and reproducibility of this assay were both 100%. All 142 newly diagnosed AML MR patients enrolled in this study received the 78 gene targeted sequencing at diagnosis as part of routine clinical workup. Somatic mutations were classified according to the 2017 guidelines for myeloid neoplasms from the Association for Molecular Pathology, American Society of Clinical Oncology, and College of American Pathologists [11]. Only pathogenic or likely pathogenic somatic mutations (equivalent to Class I and Class II variants in the gene report) were included in our mutation frequency analysis. Other baseline characteristics were collected at the same time, including age, sex, prior MDS, prior MDS/MPN, dysplasia, prior HMA exposure, ELN 2022 cytogenic categories, ELN 2024 risk categories for patients receiving less‐intensive therapies, and [11], allo‐HSCT in the following treatment.

### 2.3 Outcomes

Response assessments including CR, CR with incomplete recovery of blood counts (CRi), morphologic leukemia‐free state (MLFS), and measurable residual disease (MRD) negativity were carried out according to ELN 2022 guidelines [10]. MRD was uniformly assessed by multiparameter flow cytometry in all three cohorts. The sensitivity threshold for MRD negativity was 10^−4^ (0.01%) in all patients. MRD was evaluated for each patient after each cycle of induction therapy until the best response was achieved. After remission was achieved, MRD was also regularly monitored to detect any relapse. For those who proceeded to allo‐HSCT, MRD was also assessed pretransplant. MRD assessment was performed with identical frequency, timing, and methodology across the LIC, IC, and VEN/HMA cohorts. The overall response rate (ORR) was defined as the rate of CR/CRi/MLFS. Event‐free survival (EFS) was defined from the time of treatment initiation to refractory disease, progression, or death. OS was defined as the time from treatment initiation to death. Refractory disease was defined as failure to achieve CR or CRi at the response landmark (after 2 courses of intensive induction treatment or 180 d after commencing less‐intensive therapy). Disease progression was defined as ≥ 20% increase in bone marrow blasts, unequivocal enlargement of extramedullary disease, or clear clinical deterioration compared with the baseline or best response, assessed at any time during frontline treatment. Patients were not censored at allo‐HSCT; they were followed continuously after transplantation until an event occurred for EFS or until death for OS. The date of last follow‐up was December 31, 2023. Patients who were lost to follow‐up were censored at the date of last documented contact in both EFS and OS analyses.

### 2.4 Statistical Analysis

Absolute numbers and percentages were used for categorical variables, and differences between groups were analyzed with the Chi‐square test or Fisher's exact test. Medians and ranges were used for continuous variables and differences between groups were analyzed with the Wilcoxon Mann Whitney test. EFS and OS were evaluated by the Kaplan–Meier method with the log‐rank test. Cox regression analyses were conducted to assess the predictors for EFS and OS. We first performed univariate analyses for each candidate variable and variables with a threshold of *p* < 0.1 were then entered into the multivariable model simultaneously. 95% confidence intervals (CIs) were used to estimate hazard ratios (HRs). Survival analyses (Kaplan–Meier, log‐rank test, and Cox regression) were performed using the SPSS survival analysis module (Life Tables and Cox Regression procedures). The proportional hazards assumption was formally checked for all Cox models and was met. All statistical tests were two‐sided, and *p* ≤ 0.05 was considered statistically significant. GraphPad Prism was used for graphing.

## 3 Results

### 3.1 Patient Characteristics

**FIGURE 1:**
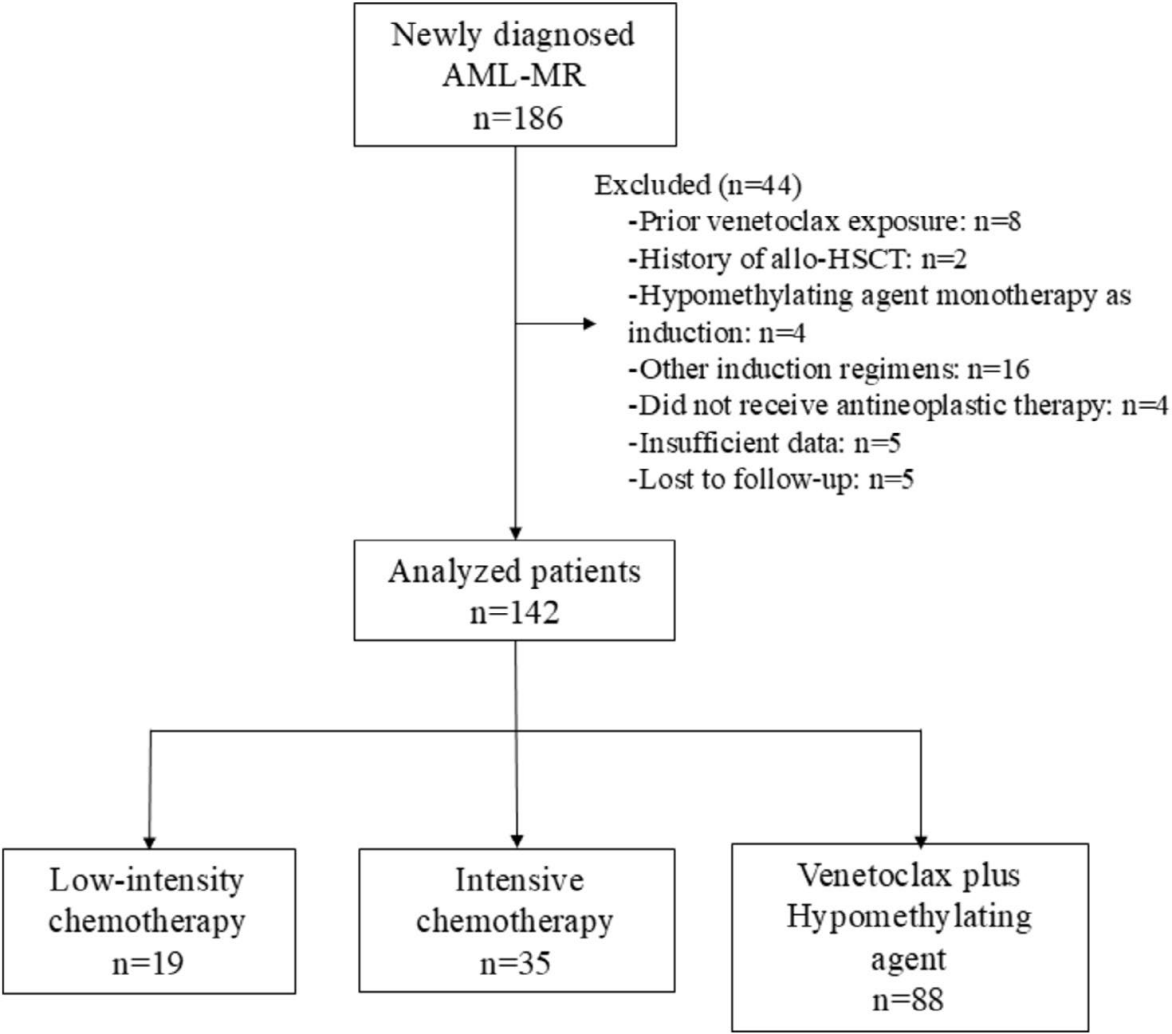
Study flow diagram of patient enrollment. allo‐HSCT: allogeneic hematopoietic stem cell transplantation; AML‐MR: acute myeloid leukemia, myelodysplasia‐related.

**FIGURE 2:**
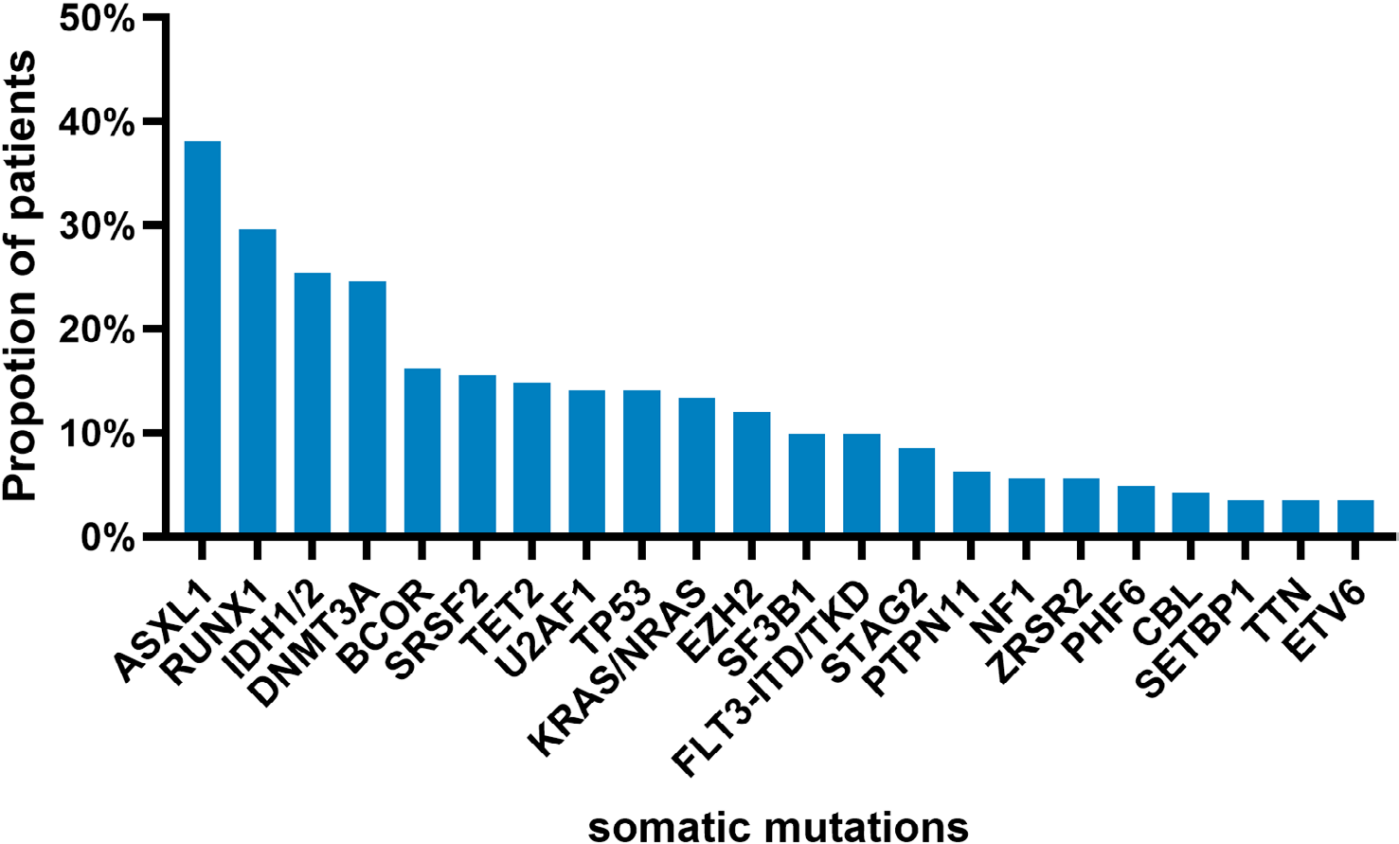
Somatic mutations in patients with acute myeloid leukemia, myelodysplasia‐related.

Between January 2019 and June 2023, 142 newly diagnosed AML‐MR patients were enrolled in the study, with a median age of 66 years (range 18–89). The flow diagram for patient enrollment in this study is presented in Figure 1. Among the 142 patients, 18 (12.7%) underwent fluorescence in situ hybridization analysis. The distribution of patients based on the AML‐MR defining criteria (WHO 2022) is presented in Table S1. The most frequent mutation in our cohort was the ASXL1 mutation, and Figure 2 shows the profile of mutations with higher probability of occurrence. The baseline characteristics are shown in Table 1. A total of 19 patients received frontline LIC, 35 patients received frontline IC, and 88 patients received frontline VEN/HMA. Frontline therapy regimens and patient distribution in each group are shown in Table S2. Patients treated with IC were generally younger than patients in the other treatment groups, and more underwent subsequent allo‐HSCT. Patients treated with VEN/HMA were more likely to have a history of MDS and HMA exposure. Other characteristics were similar between the three cohorts. Baseline characteristics and outcomes of patients excluded due to single‐agent HMA induction therapy are presented in Table S3.

**TABLE 1:** Baseline patient characteristics.

	Overall (*n* = 142)	LIC cohort (*n* = 19)	IC cohort (*n* = 35)	VEN/HMA cohort (*n* = 88)	*p*
Age					
Median	66 (18–89)	69 (31–83)	54 (18–78)	68 (38–89)	< 0.001
< 65	63 (44.4%)	6 (31.6%)	26 (74.3%)	31 (35.2%)	< 0.001
≥ 65	79 (55.6%)	13 (68.4%)	9 (25.7%)	57 (64.8%)	
Sex					
Male	87 (61.3%)	16 (84.2%)	18 (51.4%)	53 (90.2%)	0.058
Female	55 (38.7%)	3 (15.8%)	17 (48.6%)	35 (39.8%)	
Progressing from MDS	33 (23.3%)	3 (15.8%)	3 (8.6%)	27 (30.7%)	0.022
Progressing from MDS/MPN	5 (3.5%)	1 (5.3%)	0 (0.0%)	4 (4.5%)	0.509
Dysplasia	42 (29.6%)	4 (21.1%)	6 (17.1%)	32 (36.4%)	0.133
Prior HMAs exposure	27 (19.0%)	3 (15.8%)	2 (5.7%)	22 (25.0%)	0.045
ELN 2022 cytogenic categories					0.477
Intermediate	110 (81.5%)	14 (73.7%)	30 (85.7%)	73 (83.0%)	
Adverse	25 (18.5%)	5 (26.3%)	5 (14.3%)	15 (17.0%)	
ELN 2024 lessiIntensive					0.340
Favorable	87 (61.3%)	11 (57.9%)	21 (60.0%)	55 (62.5%)	
Intermediate	35 (24.6%)	5 (26.3%)	12 (34.3%)	18 (20.5%)	
Adverse	20 (14.1%)	3 (15.8%)	2 (5.7%)	15 (17.0%)	
Somatic mutations					
FLT3‐ITD/TKD mutation	14 (9.9%)	2 (10.5%)	4 (11.4%)	8 (9.1%)	0.920
IDH1/2 mutation	36 (25.4%)	7 (36.8%)	8 (22.9%)	21 (23.9%)	0.479
TP53 mutation	20 (14.1%)	3 (15.8%)	2 (5.7%)	15 (17.0%)	0.292
KRAS/NRAS mutation	19 (13.4%)	1 (5.3%)	5 (14.3%)	13 (14.8%)	0.676
RUNX1 mutations	42 (29.6%)	3 (15.8%)	11 (31.4%)	28 (31.8%)	0.367
DNMT3A	35 (24.6%)	5 (26.3%)	7 (20.0%)	23 (26.1%)	0.813
TET2	21 (14.8%)	0 (0.0%)	6 (17.1%)	15 (17.0%)	0.136
Allo‐HSCT	33 (23.2%)	1 (5.3%)	13 (37.1%)	19 (21.6%)	0.024

Abbreviations: allo‐HSCT, allogeneic hematopoietic stem cell transplantation; ELN, European LeukemiaNet; HMA, hypomethylating agent; IC, intensive chemotherapy; LIC, low‐intensity chemotherapy; MDS, myelodysplastic syndrome; MDS/MPN, myelodysplastic/myeloproliferative neoplasms; VEN/HMA, venetoclax plus hypomethylating agent.

### 3.2 Response and Early Death

ORR in patients treated with VEN/HMA was significantly higher than in those treated with LIC or IC (VEN/HMA vs. LIC, 72.7% vs. 42.1%, *p* = 0.010; VEN/HMA vs. IC, 72.7% vs. 54.3%, *p* = 0.049). There was no significant difference in ORR between patients in the LIC and IC cohorts. The MRD negative rate in the VEN/HMA cohort was also significantly higher than in the LIC cohort (65.9% vs. 31.6%, *p* = 0.006). The 60‐day mortality rate was similar in all cohorts (Table 2).

**TABLE 2:** Response and early death corresponding to different treatment strategies.

	LIC (*n* = 19)	IC (*n* = 35)	VEN/HMA (*n* = 88)	*p* (LIC vs. IC)	*p* (LIC vs. VEN/HMA)	*p* (IC vs. VEN/HMA)
ORR	8 (42.1%, 95% CI: 22.1%–63.3%)	19 (54.3%, 95% CI: 36.6%–71.2%)	64 (72.7%, 95% CI: 62.4%–81.5%)	0.393	0.010	0.049
MRD negative	6 (31.6%, 95% CI: 12.6%–56.6%)	19 (54.3%, 95% CI: 36.6%–71.2%)	58 (65.9%, 95% CI: 54.8%–75.7%)	0.110	0.006	0.229
60‐day mortality	2 (10.5%, 95% CI: 1.3%–33.1%)	1 (2.9%, 95% CI:0.1%–14.9%)	3 (3.4%, 95% CI: 0.7%–9.7%)	0.580	0.215	1.000

Abbreviations: IC, intensive chemotherapy; LIC, low‐intensity chemotherapy; MRD, measurable residual disease; ORR, overall response rate; VEN/HMA, venetoclax plus hypomethylating agent.

### 3.3 Survival by Treatment Strategy

**FIGURE 3:**
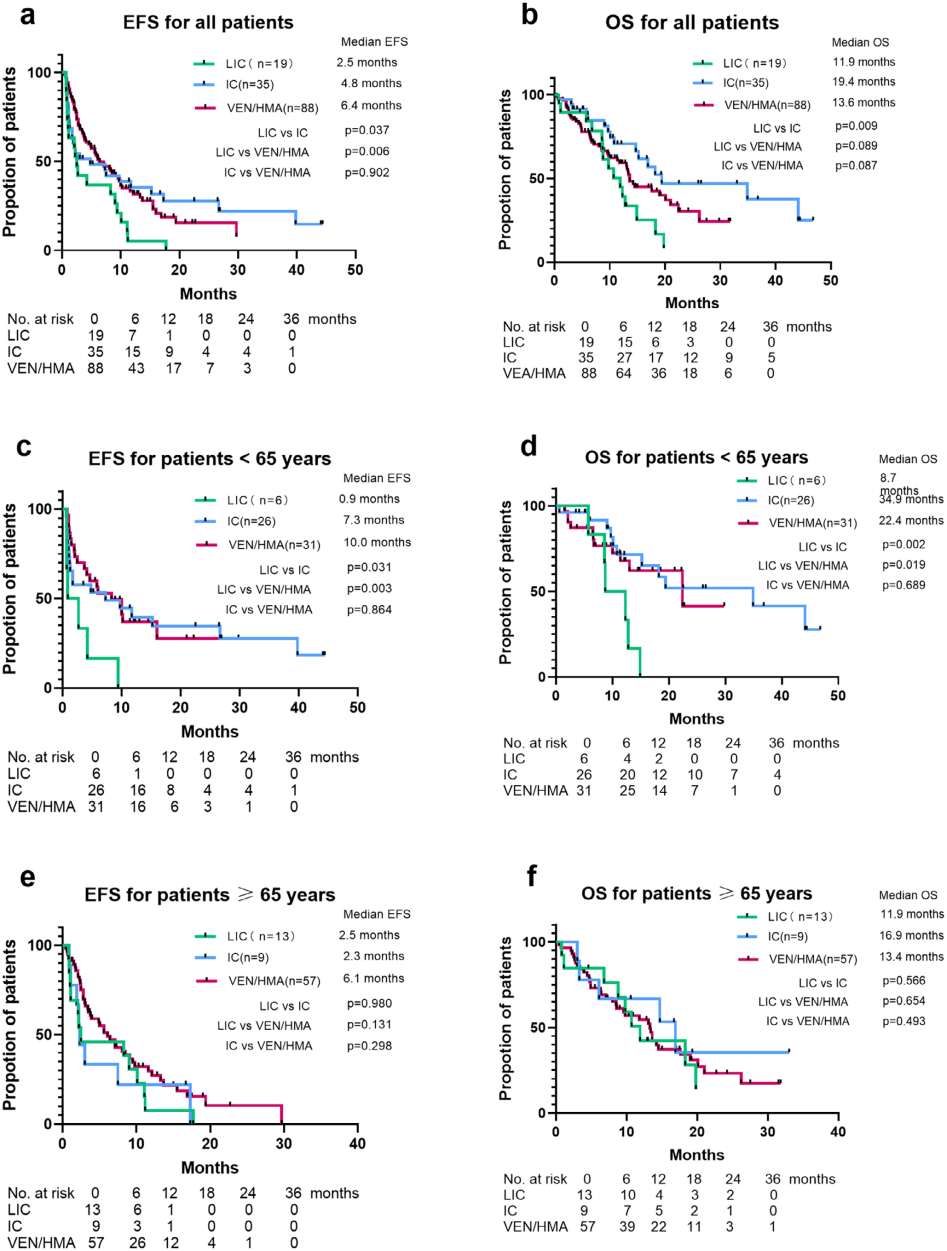
Kaplan–Meier curves for EFS and OS. (a) EFS and (b) OS in the total cohort according to treatment strategy. Panels (c) and (d) are restricted to patients < 65 years of age; panels e and f are restricted to patients ≥ 65 years of age. (c) EFS and (d) OS in patients < 65 years old. (e) EFS and (f) OS in patients ≥ 65 years old. AML‐MR: acute myeloid leukemia, myelodysplasia‐related; EFS: event‐free survival; IC: intensive chemotherapy; LIC: low‐intensity chemotherapy; OS: overall survival; VEN/HMA: venetoclax plus hypomethylating agent.

At the time of analysis, the median follow‐up duration for survivors was 14.9, 26.0, and 19.0 months in the LIC, IC, and VEN/HMA cohorts, respectively. A total of 19 EFS events and 15 deaths were observed in the LIC cohort, 26 EFS events and 16 deaths in the IC cohort, and 64 EFS events and 47 deaths in the VEN/HMA cohort. Among patients treated with LIC, the median EFS was 2.5 months, which was inferior to that of patients treated with IC (median 4.8 months, *p* = 0.037) or VEN/HMA (median 6.4 months, *p* = 0.006). The OS of patients treated with LIC was inferior to that of patients treated with IC (median 11.9 vs. 19.4 months, *p* = 0.009). No statistically significant differences in EFS or OS were observed between patients treated with IC and those treated with VEN/HMA (Figure 3a,b).

Because there was a significant difference in age between the treatment groups, we further analyzed the treatment outcomes of patients in different age groups. Among patients < 65 years old, the EFS and OS in patients treated with LIC were significantly inferior to those in patients treated with IC (median EFS, 0.9 vs. 7.3 months, *p* = 0.031; median OS, 8.7 vs. 34.9 months, *p* = 0.002) or VEN/HMA (median EFS, 0.9 vs. 10.0 months, *p* = 0.003; median OS, 8.7 vs. 22.4 months, *p* = 0.019). No significant differences were observed in EFS or OS between the IC and VEN/HMA groups (Figure 3c,d). In patients ≥ 65 years old, no significant differences were observed in EFS or OS among the three treatment groups (Figure 3e,f).

### 3.4 Univariate and Multivariate Analyses for EFS and OS

Multivariate analyses showed that prior HMA exposure (HR 1.79, 95% CI 1.13–2.83, *p* = 0.013) and frontline LIC therapy (HR 1.77, 95% CI 1.06–2.97, *p* = 0.030) were associated with inferior EFS, while allo‐HSCT (HR 0.48, 95% CI 0.29–0.81, *p* = 0.006) was associated with better EFS. Allo‐HSCT (HR 0.18, 95% CI 0.08–0.42, *p* < 0.001) was the only independent prognostic factor for OS (Table 3).

**TABLE 3:** Univariate and multivariate analyses of factors associated with EFS and OS.

Variables	EFS				OS			
	Univariate analysis		Multivariate analysis		Univariate analysis		Multivariate analysis	
	HR (95% CI)	*p*	HR (95% CI)	*p*	HR (95% CI)	*p*	HR (95% CI)	*p*
Age ≥ 65	1.36 (0.92–2.01)	0.121			1.77 (1.10–2.84)	0.019	0.71 (0.41–1.25)	0.598
Male	1.37 (0.92–2.03)	0.137			1.57 (0.97–2.53)	0.065	1.48 (0.90–2.44)	0.123
Progressing from MDS	1.22 (0.79–1.90)	0.368			1.51 (0.91–2.50)	0.110		
Progressing from MDS/MPN	1.61 (0.65–3.95)	0.303			2.19 (0.80–6.02)	0.129		
Dysplasia	1.16 (0.78–1.73)	0.472			1.22 (0.83–1.79)	0.323		
Prior HMAs exposure	1.72 (1.10–2.71)	0.019	1.79 (1.13–2.83)	0.013	1.49 (0.87–2.56)	0.150		
ELN cytogenic categories: adverse vs. intermediate	1.46 (0.90–2.35)	0.124			2.75 (1.63–4.63)	< 0.001	1.89 (0.95–3.75)	0.071
FLT3 ITD/TKD mutation	1.68 (0.94–3.02)	0.081	1.44 (0.80–2.59)	0.229	1.12 (0.56–2.24)	0.757		
IDH1/2 mutation	0.78 (0.50–1.22)	0.273			0.93 (0.56–1.53)	0.771		
TP53 mutation	1.22 (0.71–2.07)	0.473			2.29 (1.30–4.04)	0.004	1.87 (0.88–3.95)	0.102
KRAS/NRAS mutation	0.91 (0.50–1.65)	0.747			1.21 (0.62–2.35)	0.586		
RUNX1 mutations	1.12 (0.73–1.71)	0.602			1.25 (0.77–2.03)	0.376		
DNMT3A	0.94 (0.61–1.47)	0.792			0.93 (0.56–1.57)	0.797		
TET2	0.80 (0.47–1.37)	0.422			1.25 (0.69–2.28)	0.463		
Allo‐HSCT	0.42 (0.25–0.69)	0.001	0.48 (0.29–0.81)	0.006	0.23 (0.12–0.47)	< 0.001	0.18 (0.08–0.42)	< 0.001
LIC	1.99 (1.21–3.28)	0.007	1.77 (1.06–2.97)	0.030	1.86 (1.05–3.30)	0.033	1.18 (0.64–2.16)	0.598

Abbreviations: allo‐HSCT, allogeneic hematopoietic stem cell transplantation; CI, confidence interval; EFS, event‐free survival; ELN, European LeukemiaNet; HMA, hypomethylating agent; HR, hazard ratio; LIC, low‐intensity chemotherapy; MDS/MPN, myelodysplastic/myeloproliferative neoplasms; OS, overall survival.

### 3.5 Subgroup Analyses in Patients Treated With IC or VEN/HMA

Although there was no significant difference in survival between IC and VEN/HMA in the overall population, exploratory subgroup analyses were performed to investigate potential heterogeneity of treatment effects across key clinical and genetic subgroups with respect to EFS and OS. For EFS, a numerical trend favoring VEN/HMA was observed among patients with RUNX1 mutation (HR 0.26, 95% CI 0.12–0.59, *p* = 0.003; Figure S1a). For OS, a numerical trend favoring IC was identified in the ELN adverse cytogenetic category (HR 4.70, 95% CI 1.03–21.36, *p* = 0.019; Figure S1b). These findings should be regarded as hypothesis‐generating and exploratory, given the lack of formal treatment‐by‐subgroup interaction testing and the limited sample size within subgroups.

## 4 Discussion

The latest edition of the WHO classification redefined the diagnostic criteria for AML‐MR, emphasizing the role of genetic features associated with MDS in the diagnosis rather than morphological abnormalities. A diagnosis of AML‐MR requires one or more molecular and cytogenetic abnormalities or a history of MDS or MDS/MPN. To our knowledge, this study is the first to report the clinical features and impact of the frontline treatment approach on the outcomes of AML‐MR, especially including a large number of cases treated with VEN/HMA.

In our cohort, the median age of patients with AML‐MR was 66 years, younger than previously reported for AML‐MRC. A history of MDS or MDS/MPN was found in 26.8% of the patients, which was similar to previous reports of AML‐MRC [12, 13]. In our cohort, the majority of patients had intermediate‐risk cytogenetics, while previous studies reported that the majority of AML‐MRC patients had high‐risk cytogenetics [12, 13, 14]. This may be related to changes in diagnostic criteria. In accordance with new diagnostic criteria of AML‐MR and the development of next‐generation sequencing, we enrolled more patients with a diagnosis based on the eight MDS‐related somatic mutations, rather than MDS‐related cytogenetics. Furthermore, fluorescent in situ hybridization testing was not performed routinely in every patient in our study, which may have led to some selection bias. By definition, patients with AML‐MR have a high frequency of the eight MDS‐related gene mutations, and ASXL1 was found to be the most frequent mutation, accounting for 38% of the total population, which is similar to previous reports for AML‐MRC [7, 15]. Mutations in the RUNX1, IDH1/2, DNMT3A, TET2, and TP53 genes were also found frequently in our study.

Treatment of AML has remained largely unchanged for decades and consists mainly of IC with a combination of cytarabine and anthracycline (“7 + 3” regimen) [16]. However, CR rates in patients with AML‐MRC are reported to be low, usually between 30% and 50% [8, 12, 17]. For many older, unfit patients with AML‐MRC, less intensive regimens including single HMAs and low‐dose cytarabine therapies are reported to result in CR rates between 20% and 40% [12, 13, 18, 19]. The median OS for AML‐MRC patients is only 9–12 months even in patients that can tolerate intensive induction chemotherapy [4, 12, 19, 20]. Venetoclax has recently been approved for AML patients ≥ 75 years old, and the combination of venetoclax with HMAs shows promising efficacy and has been adopted as a new standard for older or unfit patients [9, 21, 22, 23]. A randomized phase 3 trial VIALE‐A study showed that there was a nonsignificant improvement in OS with azacitidine plus venetoclax compared with azacitidine plus placebo in patients with AML‐MRC [22]. However, this study excluded patients with any prior treatment for MDS, including HMAs, which constitutes a large subset of patients with AML‐MRC. The outcomes of patients with AML‐MR based on different treatment regimens have not been reported previously. In this study, we compared the effects of LIC, IC, and VEN/HMA in patients with AML‐MR. The results showed that the response rate in patients treated with frontline VEN/HMA was significantly higher than in patients treated with IC or LIC. Patients treated with frontline LIC had significantly worse EFS and OS than those treated with IC or VEN/HMA, especially in patients < 65 years old. No statistically significant differences in EFS or OS were observed between patients treated with VEN/HMA or IC. In multivariable analysis, LIC was also found to be the risk factor for adverse EFS. These data suggest that VEN/HMA improves outcomes in frail patients with AML‐MR compared with LIC. We observed no significant differences in survivals between VEN/HMA and IC in both older and younger patients with AML‐MR; however, the study was not designed or powered to demonstrate noninferiority, and residual confounding cannot be excluded. Prospective validation is needed before treatment selection can be influenced.

CPX‐351, a liposomal encapsulation of cytarabine and daunorubicin, is the only therapy approved for AML‐MRC and t‐AML to date [8, 24]. The phase 3, randomized, open‐label trial involving patients with de novo AML‐MRC, secondary AML, or t‐AML, showed that compared with patients treated with 7 + 3 therapy, patients treated with CPX‐351 had an improved response rate (47.7% vs. 33.3%, *p* = 0.016) and median OS (9.56 vs. 5.95 months, HR 0.69) [8]. In a retrospective study involving 50 patients with secondary AML, CR rates and OS were similar in patients treated with CPX‐351 versus patients treated with VEN/HMA [25]. In the subgroup of AML arising from antecedent hematologic disorder without prior HMA exposure, CR rates were higher in patients treated with HMA/VEN than in those treated with CPX‐351 (87% vs. 42%, *p* = 0.009). However, the sample size in the subgroup analysis was small, so the accuracy of the statistical result may be questionable. Unfortunately, CPX‐351 is not currently available in China, and its efficacy in the Chinese population cannot be assessed at this time. We look forward to promising results from CPX‐351 in future clinical studies on Chinese patients.

Allo‐HSCT has been proven to potentially overcome the adverse effect of poor prognostic factors on outcomes. Several studies have shown that the outcomes of patients with AML‐MRC and non‐AML‐MRC after allo‐HSCT were similar, and multivariate analysis did not find AML‐MRC to be an independent prognostic factor after allo‐HSCT [26, 27]. In this study, allo‐HSCT was identified as the only independent prognostic factor associated with superior OS in multivariable model (HR 0.18, 95% CI 0.08–0.42, *p* < 0.001), underscoring its critical role in improving long‐term outcomes for patients with AML‐MR. These findings highlight that frontline treatment strategies should be selected to preserve subsequent transplant eligibility in both IC‐treated and VEN/HMA‐treated patients. The consistent protective effect of allo‐HSCT on both EFS and OS supports the importance of transplant‐intent approaches in the management of AML‐MR.

There are several limitations of this study. First, this was a retrospective, two‐center study and thus inherently subject to selection bias and residual confounding that cannot be fully eliminated. Second, fluorescent in situ hybridization testing was not performed routinely, with only 18 patients undergoing combined karyotyping and fluorescent in situ hybridization testing. This incomplete cytogenetic characterization may have led to under‐ascertainment of MDS‐associated chromosomal aberrations in a subset of patients, potentially influencing the precise classification of AML‐MR according to the WHO 2022 criteria. Third, the overall sample size was modest, especially in the LIC cohort (*n* = 19), which reduces statistical power and generalizability. Fourth, data on performance status and comorbidities were not systematically collected, which may represent important unmeasured confounders. Fifth, significant baseline imbalances existed across the three treatment groups, including differences in age, prior MDS/HMA exposure, and allo‐HSCT rates. These confounding factors may limit the causal interpretation of treatment comparisons. Sixth, although treatment strategies were categorized into three groups, there remained substantial heterogeneity in treatment regimens within each cohort. Seventh, MRD assessment was not uniform across patients and centers, which may affect response evaluation. Eighth, multiple pairwise treatment comparisons (LIC vs. IC, LIC vs. VEN/HMA, and IC vs. VEN/HMA) were conducted across multiple endpoints without formal multiplicity adjustment, which may increase the risk of type I error. Ninth, allo‐HSCT was not treated as a time‐dependent covariate, and no landmark analysis was conducted, which may introduce immortal time bias and affect the estimated effect of transplantation on survival. Tenth, subgroup analyses were exploratory in nature and not adjusted for multiple comparisons; formal treatment‐by‐subgroup interaction tests were not performed. Therefore, observed differences should be interpreted as hypothesis‐generating rather than definitive evidence of differential treatment efficacy and are not sufficiently robust to guide clinical practice. Finally, external validation in independent cohorts was lacking. Future prospective studies with larger sample sizes, more comprehensive clinical data and external validation are warranted to confirm our findings.

## 5 Conclusions

Our study suggests that in patients with AML‐MR, frontline therapies with VEN/HMA or IC improve outcomes compared with LIC. Allo‐HSCT is encouraged for all eligible patients with AML‐MR.

## Author Contributions

**Peiying Fang:** investigation, methodology. **Donghua He:** data curation, writing – review and editing. **Qianqian Yang:** resources, investigation. **Yi Li:** methodology, formal analysis, writing – original draft. **Dian Jin:** writing – original draft, methodology, formal analysis. **Xinyi meng:** writing – original draft, methodology, formal analysis. **Jing Le:** resources, investigation. **Wenxiu Shu:** investigation, resources. **Zhen Cai:** funding acquisition, conceptualization. **Jingsong He:** conceptualization, funding acquisition. **Jintao Lin:** investigation, resources.

## Funding

This work was funded by the National Natural Science Foundation of China (81770217, 81872322), the Natural Science Foundation of Zhejiang Province (LY22H080003, LQ22H080008), and the Medical and Health Research Project of Zhejiang Province (2025KY1265). The funders had no role in the study design, data collection, analysis, interpretation, manuscript writing, or the decision to publish the results.

## Ethics Statement

Approval of the research protocol by an Institutional Reviewer Board: The study was approved by the Ethical Review Committee of The First Affiliated Hospital of Zhejiang University School of Medicine (approval No. IIT20230774A) and the Ethical Review Committee of The Affiliated Li Huili Hospital of Ningbo University (approval No. KY2023PJ351). All methods used in this study follow the principles of the Declaration of Helsinki. Because this is a retrospective study, the requirement for written informed consent was waived.

## Conflicts of Interest

The authors declare no conflicts of interest.

## Supporting information


**Figure S1:** Predictors favoring IC or VEN plus HMA.

**Table S1:** Distribution of patients according to AML‐MR defining criteria (WHO 2022).

**Table S2:** Frontline therapy regimens and patient distribution.

**Table S3:** Baseline characteristics and outcomes of patients excluded due to single‐agent HMA induction therapy.

## Data Availability

The data that support the findings of this study are available from the corresponding author upon reasonable request.
