# Supplementary figures and images for: Impact of Frontline Treatment Strategies on Outcomes in Patients With Acute Myeloid Leukemia, Myelodysplasia‐Related
Source: Cancer Rep (Hoboken). 2026 Jun 21;9(6):e70607. doi: 10.1002/cnr2.70607 (PMC13284085; doi:10.1002/cnr2.70607)

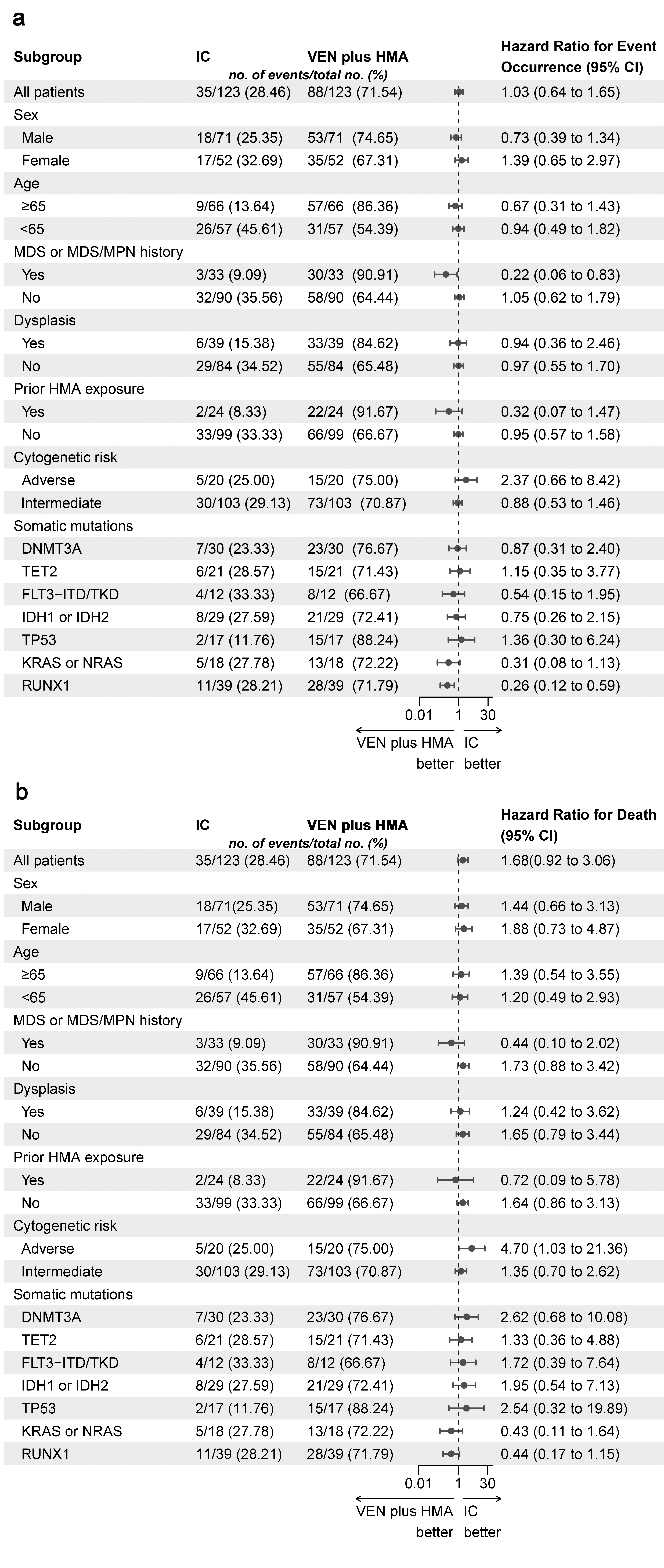

Supplement: Supplementary file 1 — Figure S1: Predictors favoring IC or VEN plus HMA. [file CNR2-9-e70607-s003.tif]
