# Supplementary material for: Impact of Frontline Treatment Strategies on Outcomes in Patients With Acute Myeloid Leukemia, Myelodysplasia‐Related
Source: Cancer Rep (Hoboken). 2026 Jun 21;9(6):e70607. doi: 10.1002/cnr2.70607 (PMC13284085; doi:10.1002/cnr2.70607)
Supplement: Supplementary file 2 — Table S1: Distribution of patients according to AML‐MR defining criteria (WHO 2022). [file CNR2-9-e70607-s004.docx]

Table S1. Distribution of patients according to AML-MR defining criteria (WHO 2022)

| AML-MR diagnostic criterion | n | % |
| --- | --- | --- |
| Mutation-defined only (8 MDS-related genes only) | 84 | 59.2 |
| Cytogenetic-defined only | 13 | 9,2 |
| Prior MDS/MDS-MPN only | 7 | 4.9 |
| Combination subgroups | 38 | 26.8 |
| Mutation + Cytogenetic | 8 | 5,6 |
| Mutation + Prior MDS/MDS-MPN | 23 | 16.2 |
| Cytogenetic + Prior MDS/MDS-MPN | 3 | 2.1 |
| Mutation + Cytogenetic + Prior MDS/MDS-MPN | 4 | 2.8 |
