# Supplementary material for: Impact of Frontline Treatment Strategies on Outcomes in Patients With Acute Myeloid Leukemia, Myelodysplasia‐Related
Source: Cancer Rep (Hoboken). 2026 Jun 21;9(6):e70607. doi: 10.1002/cnr2.70607 (PMC13284085; doi:10.1002/cnr2.70607)
Supplement: Supplementary file 3 — Table S2: Frontline therapy regimens and patient distribution. [file CNR2-9-e70607-s002.docx]

Table S2. Frontline therapy regimens and patient distribution

| Frontline therapies | N | % |
| --- | --- | --- |
| Low-intensity chemotherapy | 19 |  |
| low dose cytarabine | 1 | 5.3 |
| low dose cytarabine + anthracycline | 3 | 15.8 |
| HMA based regimens | 15 | 78.9 |
| Intensive chemotherapy | 35 |  |
| cytarabine + daunorubicin/idarubicin | 21 | 60.0 |
| cytarabine + homoharringtonine ± aclacinomycin | 8 | 22.9 |
| HMA+ cytarabine + daunorubicin/idarubicin | 2 | 5.7 |
| HMA+ cytarabine + homoharringtonine ± aclacinomycin | 4 | 11.4 |
| VEN/HMA | 88 |  |
| Azacitidine + venetoclax | 84 | 95.5 |
| Decitabine + venetoclax | 4 | 4.5 |
