# Supplementary material for: Impact of Frontline Treatment Strategies on Outcomes in Patients With Acute Myeloid Leukemia, Myelodysplasia‐Related
Source: Cancer Rep (Hoboken). 2026 Jun 21;9(6):e70607. doi: 10.1002/cnr2.70607 (PMC13284085; doi:10.1002/cnr2.70607)
Supplement: Supplementary file 4 — Table S3: Baseline characteristics and outcomes of patients excluded due to single‐agent HMA induction therapy. [file CNR2-9-e70607-s001.docx]

Table S3. Baseline characteristics and outcomes of patients excluded due to single-agent HMA induction therapy.

|  | Patient 1 | Patient 3 | Patient 3 | Patient 4 |
| --- | --- | --- | --- | --- |
| Age(yeas) | 52 | 73 | 71 | 60 |
| Sex | female | male | female | female |
| Progressing from MDS | yes | yes | no | yes |
| Progressing from MDS/MPN | no | no | no | no |
| dysplasia | no | yes | no | yes |
| Prior HMAs exposure | no | no | no | no |
| ELN 2022 cytogenic categories | adverse | adverse | intermediate | intermediate |
| ELN 2024 Less-Intensive | favorable | favorable | adverse | favorable |
| Somatic mutations | IDH2 | IDH1 | ASXL1, TP53, DDX41, EZH2 | ASXL1, RUNX1, EZH2, ZRSR2, TTN |
| Allo-HSCT | no | no | no | no |
| Response | SD | PD | CR | PR |
| EFS (months) | 2.2 months | 0.7 month | 12.2 months | 2.8 months |
| OS (months) | 16.5(alive, censored) | 8.4(dead) | 22.8(alive, censored) | 27.8(alive, censored) |
